# Health-related quality of life among the elderly with heart failure: a generic measurement

**DOI:** 10.1590/S1516-31802010000400003

**Published:** 2010-07-01

**Authors:** Izabel Cristina Ribeiro da Silva Saccomann, Fernanda Aparecida Cintra, Maria Cecilia Bueno Jayme Gallani

**Affiliations:** I RN, MD. Nursing Department, Medical and Biological Sciences Center, Pontifícia Universidade Católica (PUC), Sorocaba, São Paulo, Brazil.; II RN, PhD. Nursing Department, School of Medical Sciences, Universidade Estadual de Campinas (Unicamp), Campinas, São Paulo, Brazil.

**Keywords:** Aged, Aging, Heart failure, Quality of life, Questionnaires, Idoso, Envelhecimento, Insuficiência cardíaca, Qualidade de vida, Questionários

## Abstract

**CONTEXT AND OBJECTIVE:**

Health-related quality-of-life (HRQoL) instruments have been greatly used among patients with heart failure (HF), although few of them are specific for elderly people. Among the generic instruments, the Medical Study 36-item Short-Form Health Survey (SF-36) is widely used. The aim here was to evaluate HRQoL among elderly individuals with HF through this generic instrument.

**DESIGN AND SETTING:**

Cross-sectional study at two university hospitals in São Paulo, Brazil.

**METHODS:**

170 elderly people with HF who were being followed up as outpatients were interviewed. To evaluate HRQoL, SF-36 was used.

**RESULTS:**

The sample was composed of subjects with a mean age of 67.5 (± 6.2) years, with a diagnosis of HF for 65.9 (± 42.4) months, in functional class I (38.8%; 66) or II (42.9%; 73) and with reduced left ventricular ejection fraction (LVEF) (51.2%). The mental and social HRQoL domains did not seem to be compromised, since they presented high scores. Patients with HF typically had impaired physical capacity, which may explain the lower scores in the physical domain. Cronbach's alpha coefficients were greater than 0.77 for all dimensions, except for general health status.

**CONCLUSION:**

The HRQoL measurements using SF-36 presented a high level of reliability when applied to Brazilian elderly individuals with HF. This population presented lower scores for the functional capacity and physical dimensions. This provides support for intervention studies aiming towards optimization of HRQoL in this group.

## RESUMO

**CONTEXTO E OBJETIVO:**

Instrumentos de qualidade de vida relacionada à saúde (QVRS) têm sido muito utilizados em pacientes com Insuficiência Cardíaca (IC), embora poucos sejam específicos a pessoas idosas. Entre os instrumentos genéricos, o Medical Study 36-item Short-Form Health Survey (SF-36) é utilizado com frequência. O objetivo foi avaliar a QVRS em idosos portadores de IC por meio de um instrumento genérico.

**TIPO DE ESTUDO E LOCAL:**

Estudo transversal realizado em dois hospitais universitários de São Paulo, Brasil.

**MÉTODOS::**

Foram entrevistados 170 idosos com insuficiência cardíaca e em acompanhamento ambulatorial. Para avaliar a QVRS, foi utilizado o SF-36.

**RESULTADOS:**

A amostra foi composta por sujeitos com média de idade de 67,5 (± 6.2) anos, com tempo médio de IC de 65,9 (± 42.4) meses, em classe funcional I (38,8%, 66) ou II (42,9%, 73) e fração de ejeção do ventrículo esquerdo (FEVE) rebaixada (51,2%). Os domínios da QVRS que avaliam os aspectos mental e social não parecem estar comprometidos, uma vez que apresentaram altas pontuações. Pacientes com insuficiência cardíaca geralmente têm a capacidade física prejudicada, o que pode justificar a pontuação mais baixa no domínio físico. Os coeficientes alfa de Cronbach foram superiores a 0,77 para todas as dimensões, exceto Estado Geral de Saúde.

**CONCLUSÃO:**

A medida da QVRS pelo SF-36 apresentou alto nível de confiabilidade quando aplicado em idosos brasileiros com IC. Essa população apresentou escores mais baixos nas dimensões capacidade funcional e aspectos físicos, fornecendo subsídios para estudos de intervenções que contribuam para a otimização da QVRS desse grupo.

## INTRODUCTION

Aging is one of the greatest challenges for healthcare. Attending the elderly is already a major public health problem, not only in developed countries, but also in developing countries like Brazil.^1^

One of the consequences of aging societies is the growing prevalence of chronic diseases, particularly cardiovascular diseases.^2^ Among these, heart failure (HF) has become one of the most prevalent, and the trend is for its prevalence to increase further over coming decades, along with the aging process.^3,4^ Among the elderly, diagnosing HF is more difficult because of limitations of perception and self-limitation of physical activities, in addition to hearing, memory and comprehension losses, which are all very common in this age range. Thus, these patients are physically, psychologically and socially affected.^5^ Nearly 90% of such patients present dyspnea and fatigue,^6,7^ and emotional symptoms like depression and anxiety are also likely.^5^ These signs and symptoms cause limitations to patients’ daily lives, especially regarding physical activity, and lead to constant hospitalization.^8,9^

Within this context, symptom relief takes on a vital role that is even more important than extending life itself.^10^

Evaluating individuals’ quality of life as they get older has become increasingly important over recent decades, with increasing life expectancy.^11^ Such evaluations are based on the subjects’ perception of their own health status, which is influenced by the cultural context.^12^ Therefore, evaluating health-related quality of life (HRQoL) among elderly individuals suffering from HF is very important, especially in relation to disease perception and the way in which the disease interferes with their lives.

HRQoL is a broad concept that is often described as subjective and multidimensional, including physical, psychological and social dimensions. In the literature, HRQoL questionnaires differ conceptually, regarding the subjective and objective aspects of choosing the items, nature and weights of each domain.^13^ Both generic and specific instruments have the aim of objectively measuring dimensions that are considered subjective, thereby making quality-of-life measurement possible.^12^

The options for the measurement tool should be based on the objectives of the study and on the availability of the instrument (developed or culturally adapted) for the target population. Analysis on the psychometric properties of an instrument is critical for evaluating its reliability and the HRQoL dimensions for specific groups, like the elderly.^14^ In addition, the components of the instrument should be clear, the population and the disease for which the measurement was developed should be defined, and the instrument has to be simple and straightforward, such that the time taken to administer it is appropriate.^12^

Among the instruments for HF that are currently used, the Medical Study 36-item Short-Form Health Survey (SF-36) is one of the most frequently applied generic instruments for adult populations.^15-17^ Given the importance of applying this instrument to elderly populations, the objective of this study was to evaluate HRQoL among elderly individuals with HF using a generic measurement tool.

## METHODS

A convenience sample of 170 elderly clinical outpatients with a medical diagnosis of HF was gathered from two university hospitals in the state of São Paulo, Brazil. The age of 60 or over was used to define elderly adults, as outlined in the recommendations of the World Health Organization (WHO) for defining elderly people in developing countries.^18^ The determination of the sample size, along with the eligibility and exclusion criteria, are described elsewhere.^14^

### Data collection

Data collection was performed by the first author between June and November 2005. The first step was analysis of the medical files to determine eligible patients. The researcher met with patients before their routine medical appointments, explained the purpose of the study and invited them to participate. All patients involved in the study signed an informed consent form. The ethics committee of each institution approved the study.

Data referring to patients’ clinical condition was gathered through analyses of the medical files: HF etiology, length of time with HF diagnosis, left ventricular ejection fraction (LVEF), New York Heart Association class assessment (NYHA); HF symptoms (dyspnea, fatigue, angina and palpitation) and comorbidities. Thereafter, the patients were interviewed, which included asking demographic questions (age, gender, race, marital status, schooling, work situation and family income) and applying the SF-36. The researcher read out all the questions to the patients in order to assist patients with low reading skills or visual handicaps.

### Instrument

The instrument used was the Medical Outcomes Study (MOS) 36-item Short-Form Health Survey (SF-36 version 1), Brazilian version, adapted by Ciconelli.^19^ This is a generic HRQoL instrument comprising 36 items, including eight scales that measure: physical functioning (PF) (10 items), role-physical (RP) (4 items), pain (P) (2 items), general health perception (GHP) (5 items), vitality (VT) (4 items), social functioning (SF) (2 items), role-emotional (RE) (3 items), mental health (MH) (5 items) and one question of comparative evaluation, comparing the current health condition to the health condition one year before the interview. Each dimension is individually analyzed, and the scores for the eight components may range from 0 to 100, with higher scores indicating better HRQoL.^19^

### Statistical analysis

The data were initially transferred to a spreadsheet in the Excel Windows 98 software and afterwards to SAS for Windows (Statistical Analysis System), version 8.02. Descriptive statistics (means, standard deviations (SDs), standard errors (SEs) and ranges) were calculated for the instrument. Internal consistency was evaluated through Cronbach's alpha. The criterion a > 0.70 was established as evidence of satisfactory internal consistency.^20^

## RESULTS

The sociodemographic and clinical characteristics of the 170 patients are described in Tables 1 and 2. The sample was predominantly male (58.2%) and professionally inactive (83.0%), with a mean age of 67.5 (± 6.2) years, mean schooling level of 3.6 (± 3.4) years and monthly family income of 480.00 US dollars. The subjects presented a mean length of HF diagnosis of 65.9 (± 42.4) months. Ischemic heart disease and hypertension were the most frequent etiologies (46.5% and 32.4%). The left ventricular ejection fraction (LVEF), analyzed through echocardiogram data (Teichholtz method; n = 98) and through “first pass” gated scintigraphy (n = 66), was low in 51.2%. The majority of the elderly individuals were in functional classes I and II (38.8% and 42.9%). The most prevalent comorbidities were: systemic arterial hypertension, 77.6% (132); arterial disease, 44.1% (75); dyslipidemia, 35.9% (61); and type 2 diabetes mellitus, 34.7% (59).

**Table 1.** Sociodemographic profile of the 170 elderly people with heart failure

Variable	n (%)
**Work status**	
*Active*	
Employed	5 (2.9)
Retired and working	7 (4.1)
**Subtotal**	**12 (7.0)**
*Inactive*	
Unemployed	1 (0.6)
On sickness benefit	1 (0.6)
Retired (disability)	89 (52.4)
Retired (compulsory)	50 (29.4)
**Subtotal**	**141 (83.0)**
*Housewife*	17 (10.0)
**Gender**	
Male	99 (58.2)
Female	71 (41.8)
**Race**	
Caucasian	145 (85.3)
Non-Caucasian	25 (14.7)
**Marital status**	
*With spouse*	
Married	105 (61.8)
Living together	1 (0.6)
**Subtotal**	**106 (62.4)**
*Without spouse*	
Widow/widower	47 (27.6)
Separated	9 (5.3)
Single	8 (4.7)
**Subtotal**	**64 (37.6)**

**Table 2.** Clinical profile of the 170 elderly people with heart failure

Variable	Distribution into categories	n (%)
LVEF*	Lowered	44 (26.9)
Echocardiogram (Teichholtz) (n = 98†)	Normal	54 (32.9)
Gated scintigraphy (n = 66‡)	Lowered	40 (24.4)
	Normal	26 (15.8)
NYHA class	I	66 (38.8)
	II	73 (42.9)
	III/IV	31 (18.3)
Etiology		
	Ischemic heart disease	79 (46.5)
	Systemic arterial hypertension	55 (32.4)
	Chagas disease	25 (14.7)
	Dilated myocardiopathy	15 (8.8)
Comorbidities		
	Systemic arterial hypertension	132 (77.6)
	Ischemic disease	75 (44.1)
	Elevated serum cholesterol	61 (35.9)
	Diabetes mellitus type 2	59 (34.7)

* LVEF = left ventricular ejection fraction;

† missing data = 4;

‡ missing data = 2; NYHA = New York Heart Association assesment.

The scores in each SF-36 dimension are described in Table 3. The patients presented higher scores in the dimensions that evaluate pain, social functioning and emotional roles; medium scores in the role-physical and mental health dimensions; and lower scores in the dimensions that evaluate physical functioning, general health perception and vitality.

**Table 3.** Scores for the 36-item Short-Form Health Survey dimensions for the 170 elderly people with heart failure

Dimension	Mean	SD	Median	Actual range	Potential range
Physical functioning	51.6	20.2	51.2	6.2-95	0-100
Role-physical	64.1	44.7	100.0	0-100	0-100
Pain	70.4	30.4	72.0	0-100	0-100
General health perception	50.3	19.9	52.0	5-100	0-100
Vitality	53.7	19.9	50.0	15.0-95	0-100
Social functioning	77.4	26.9	87.5	12.5-100	0-100
Role-emotional	86.3	32.0	100.0	0-100	0-100
Mental health	65.7	21.4	72.0	4.0-100	0-100

SD = standard deviation.

The mean scores obtained through SF-36 in this study, and in other studies using the same instrument, are described in Table 4.^21-26^

**Table 4.** Mean scores for the 36-item Short-Form Health Survey dimensions in this study and in other studies

Dimension	This study	Jenkinson et al.^21^	Ekman et al.^22^	Hayes et al.^23^	Cruz^24^	Souza^26^	Middel et al.^27^
Population		Elderly people	Elderly people with HF	Adults and elderly people	Elderly people with other diseases	Elderly people with CRF	Adults and elderly people with HF
Physical functioning	51.6	34.43	39.9	44.0	38.9	49.1	56.6
Role-physical	64.1	30.74	25.3	44.6	52.3	36.0	27.3
Pain	70.4	71.77	61.8	69.2	60.5	60.0	80.9
General health perception	50.3	60.84	43.8	54.1	70.7	55.6	56.0
Vitality	53.7	50.33	35.4	50.0	60.0	56.5	48.2
Social functioning	77.4	68.49	75.4	71.0	65.6	82.7	60.4
Role-emotional	86.3	54.64	61.6	43.6	52.7	79.7	54.8
Mental health	65.7	72.98	68.9	69.1	67.4	68.6	64.6

CRF = chronic renal failure;. HF = heart failure.

SF-36 reliability was evaluated in terms of internal consistency using Cronbach's alpha coefficient. Alpha values were higher than 0.77 for all dimensions except for the general health perception dimension, in which alpha was 0.60. Table 5 presents the Cronbach's alpha values observed in this and other studies.^26-28^

**Table 5.** Cronbach's alpha for the 36-item Short-Form Health Survey (SF-36) in this study and in other studies

SF-36	This study	Brazier et al.^33^	Souza^26^	Middel et al.^27^	McHorney et al.^28^
		Elderly people		Adults and elderly people with heart failure	General population
Physical functioning	0.85	0.91	0.89	0.93	0.93
Role-physical	0.95	0.86	0.79	0.91	0.84
Pain	0.93	0.81	0.91	0.89	0.82
General health perception	0.60	0.66	0.64	0.76	0.78
Vitality	0.77	0.84	0.65	0.84	0.87
Social functioning	0.82	0.56	0.56	0.78	0.85
Role-emotional	0.93	0.83	0.80	0.90	0.83
Mental health	0.83	0.80	0.79	0.84	0.90

## DISCUSSION

The purpose of this study was to evaluate HRQoL among elderly individuals with HF, using a generic instrument. Over recent years, the literature has significantly improved in terms of the numbers of studies on quality of life in HF cases. Nevertheless, few studies have addressed the specificity of HF and its relationship with HRQoL among the elderly, especially in Brazil, using a generic HRQoL instrument.

A data survey performed in the Cochrane Library, Lilacs and Medline, from 2000 to 2009, and using the keywords “quality of life” and “heart failure” resulted in 339 abstracts: three from Cochrane, 24 from Lilacs and 312 from Medline. Of these 339 abstracts, 180 were effectively related to the present topic and were therefore selected. Among these 180 studies, the SF-36 was used in only 51 of them, either alone or in association with another instrument (which was usually specific for HRQoL). The Living with Heart Failure Questionnaire (LHFQ), which is a specific instrument, was used in 45 studies, and other HRQoL instruments were applied in 62 studies. Among the studies that used the SF-36, 33 were conducted among elderly individuals, in different contexts, which confirms the relevance of the present study.

In the literature, other generic questionnaires are available for the elderly, such as the Geriatric Quality of Life Questionnaire (WHOQOL-OLD). However, as already mentioned, the SF-36 is frequently used in Brazil and worldwide. Among the specific questionnaires, there are other instruments specific to cardiovascular diseases, but they have not been adapted for use among Brazilian elderly people.

The highest HRQoL scores were observed for the role-emotional, social functioning and mental health dimensions. In the pain dimension, the elderly subjects also had high scores, thus showing that they did not experience pain in such a way that it would impair their quality of life. There were lower scores relating to the general health perception, physical functioning and vitality dimensions. Thus, among these elderly people with HF, the greatest impairment of their quality of life was in relation to the physical domain.

Previous studies^5,21,22^ have described similar scores among elderly individuals with HF. They have also shown that elderly people present more impairment of HRQoL in the dimensions that evaluate physical health, since the subjects generally presented significant functional losses. On the other hand, a study carried out on a younger population has indicated not only physical impairment but also emotional impairment.^23^ Other studies^24-26^ on elderly populations suffering from different chronic diseases have described physical functioning and role-physical as the HRQoL dimensions most affected. In a recent study on a sample of 1958 Brazilian elderly individuals, Lima et al.^29^ observed significant social inequality in HRQoL, especially in relation to physical functioning and physical role. HRQoL was shown to be worse among: elderly women, individuals of more advanced age, those with lower incomes and lower schooling levels and those who practiced evangelical religions, in comparison with the Catholic faith.

As the disease progresses, patients usually present intolerance to physical activity, which can be explained by occurrences of respiratory discomfort, fatigue and palpitation (triggered by the incapacity of the heart to maintain sufficient cardiac debit to meet the oxygen demands), as well as by morphological and metabolic changes in the skeletal musculature. Furthermore, elderly individuals in a more advance state of HF tend to remain in a lying down position most of the time and reduce all their everyday activities.^25^ The strong association between HRQoL and symptoms suggests that there is a need for both pharmacological and non-pharmacological interventions to improve HF symptoms.^30^

It is interesting to note that, in the present study, the HRQoL domains relating to mental and social traits did not seem to be compromised. This was contrary to what was reported in another Brazilian study, which revealed that the emotional aspects of HRQoL were compromised, thus suggesting that psychological factors such as fear and anxiety could lead to introspection and depression.^25^ In the literature, it has been shown that the elderly tend to accept the limitations imposed by chronic diseases better than do younger adults, which some authors have referred to as resilience.^31^

The findings from the present study are in agreement with data in the literature, given that when subjects with HF develop functional loss, thereby limiting their daily activities, this can also compromise their HRQoL. These results should be taken into account when evaluating intervention studies, and during clinical practice.^32^

The dimension of the SF-36 for which the largest number of items remained unanswered was physical functioning. Many of these questions were not applicable to these subjects, because they performed lighter activities due to their age. Similar issues need to be taken into account when HRQoL questionnaires are used among the elderly. Another important issue is the number of options of answers to some questions in the SF-36. Many of the elderly people in the present study had difficulty in understanding the different possibilities, and the interviewer had to repeat them many times. Similar results were also obtained by Cruz and Diogo.^24^

The internal consistency of the SF-36 instrument was high, thus providing evidence of satisfactory reliability. The Cronbach's alpha coefficients in this study were similar to those reported in other investigations.^26,33^

## CLINICAL IMPLICATIONS

HF is a chronic and progressive disease, in which lower HRQoL is associated with worse clinical outcomes, including high rates of hospital readmission and mortality. The results from the present study show that there was a relationship between HRQoL and physical traits. In clinical practice, it has been observed that non-pharmacological interventions improve patients’ physical symptoms and their functional capacity. Therefore, the present study suggests that strategies for improving HF patients’ physical condition, and hence their HRQoL, should be sought.

## CONCLUSIONS

The SF-36 showed that the elderly people of the present study presented greater impairment of aspects of their quality of life relating to physical functioning and physical role. These results suggest that efforts should be made within clinical practice to improve the physical functioning of elderly individuals with HF, and hence their HRQoL.

A careful evaluation of the items that are not answered when this instrument is applied is highly recommended. The high reliability of the SF-36 among this population is an important point to be taken into account in choosing instruments for evaluating HRQoL.
